# Recovery and Analysis of Bacterial Membrane Vesicle Nanoparticles from Human Plasma Using Dielectrophoresis

**DOI:** 10.3390/bios14100456

**Published:** 2024-09-25

**Authors:** Jason P. Ware, Delaney K. Shea, Shelby L. Nicholas, Ella A. Stimson, Jessica L. Riesterer, Stuart D. Ibsen

**Affiliations:** 1 Cancer Early Detection Advanced Research Center, Knight Cancer Institute, Oregon Health and Science University, Portland, OR 97201, USAnichoshe@ohsu.edu (S.L.N.); stimson@ohsu.edu (E.A.S.); riesterj@ohsu.edu (J.L.R.); 2 Department of Biomedical Engineering, School of Medicine, Oregon Health and Science University, Portland, OR 97201, USA

**Keywords:** dielectrophoresis, bacteria-derived vesicles, liquid biopsy, plasma, diagnostics

## Abstract

Bacterial membrane vesicle (BMV) nanoparticles are secreted naturally by bacteria throughout their lifecycle and are a rich source of biomarkers from the parent bacteria, but they are currently underutilized for clinical diagnostic applications, such as pathogen identification, due to the time-consuming and low-yield nature of traditional recovery methods required for analysis. The recovery of BMVs is particularly difficult from complex biological fluids. Here, we demonstrate a recovery method that uses dielectrophoretic (DEP) forces generated on electrokinetic microfluidic chips to isolate and analyze BMVs from human plasma. DEP takes advantage of the natural difference in dielectric properties between the BMVs and the surrounding plasma fluid to quickly and consistently collect these particles from as little as 25 µL of plasma. Using DEP and immunofluorescence staining of the LPS biomarker carried on BMVs, we have demonstrated a lower limit of detection of 4.31 × 10^9^ BMVs/mL. The successful isolation of BMVs from human plasma using DEP, and subsequent on-chip immunostaining for biomarkers, enables the development of future assays to identify the presence of specific bacterial species by analyzing BMVs from small amounts of complex body fluid.

## 1. Introduction

Bacterial membrane vesicles (BMVs) provide valuable information about the bacteria that secrete them and can carry biomarkers for bacterial detection. BMVs, also known as membrane vesicles (MVs), or outer membrane vesicles (OMVs) when derived from Gram-negative bacteria specifically, are 20–300 nm diameter spherical lipid-bound nanoparticles that pinch off of bacteria and can contain proteins, nucleic acids, polysaccharides, and lipids from their parent bacteria [1,2]. BMVs are produced as a means of inter- and intra-species communication, pathogenesis, and immunomodulation and are naturally secreted throughout the bacterial lifecycle [1,3,4,5]. BMVs contain a similar membrane composition to the parent bacteria cell wall and contain proteins specific to their parent [6,7,8]. BMVs carry useful biomarkers to identify bacteria in wastewater or in food, to analyze microbiomes, to rapidly identify bacterial infections with high specificity in clinical diagnostic applications, and to monitor the treatment of infections. In particular, there is potential for using BMVs to diagnose septicemia, an infection of the blood leading to sepsis. Sepsis is a life-threatening disease causing or contributing to over 11 million deaths per year, concentrated in low- and middle-income countries, and often goes undiagnosed due to difficulties detecting and culturing the bacteria [9]. Rapid and accurate identification of the infecting bacteria is key to prescribing the correct antibiotics. Circulating BMVs are less affected by antibiotics than live bacteria, meaning that even if broad-spectrum antibiotic therapy is started immediately, the sepsis-causing strain can still be identified by BMVs left in circulation. This would allow physicians to cease the harmful delivery of unnecessary antibiotics once the strain is identified. *Neisseria meningitidis* OMVs have been shown to be stable at 4 °C for a year, although the prevalence and circulation time of BMVs in vivo during infection have not yet been elucidated [10,11].

An extensive review of in-patient sepsis found that in 70.1% of cases, a causal organism was not identified, making rapid and accurate identification a critical unmet clinical need [12]. Further, identification via BMVs eliminates the need to wait for the extended length required for blood cultures, which, despite the time investment of multiple days, may still fail [11]. In addition, BMVs could play a role in cancer detection. Not only is there evidence that host microbiotic changes can affect cancer risk, but tumors infected by bacteria can develop tumor microbiomes, the composition of which has been shown to play a crucial role in tumor growth and metastasis [13,14]. The BMVs released from the tumor into body fluids would be collectable for use as a diagnostic tool to inform treatment decisions.

Due to the valuable diagnostic biomarkers carried by BMVs, there is a broad and growing interest in developing methods to quickly and easily recover BMVs from body fluids in a manner that would be suitable for clinical translation. However, collecting BMVs from complex fluids using traditional methods is a challenge due to their small size and low buoyant density, making it not only time-consuming but also a low-yield endeavor that becomes worse with more complex clinical samples. Currently, ultracentrifugation (UC) is the gold standard for the isolation of BMVs. During BMV purification by UC, the sample is spun at high speeds to pellet BMVs; however, this takes multiple hours, and other cellular fragments and debris will end up in the pellet [15]. Density gradient purification can yield a higher purity, wherein the sample is ultracentrifuged in a tube with layered liquids of increasing density, allowing for better separation of BMVs from cellular debris based on density. Density gradient purification, however, is even more time consuming than standard UC; it dilutes the sample and is not amenable to scaling, so it cannot be translated effectively to the clinical laboratory setting.

**Figure 1:**
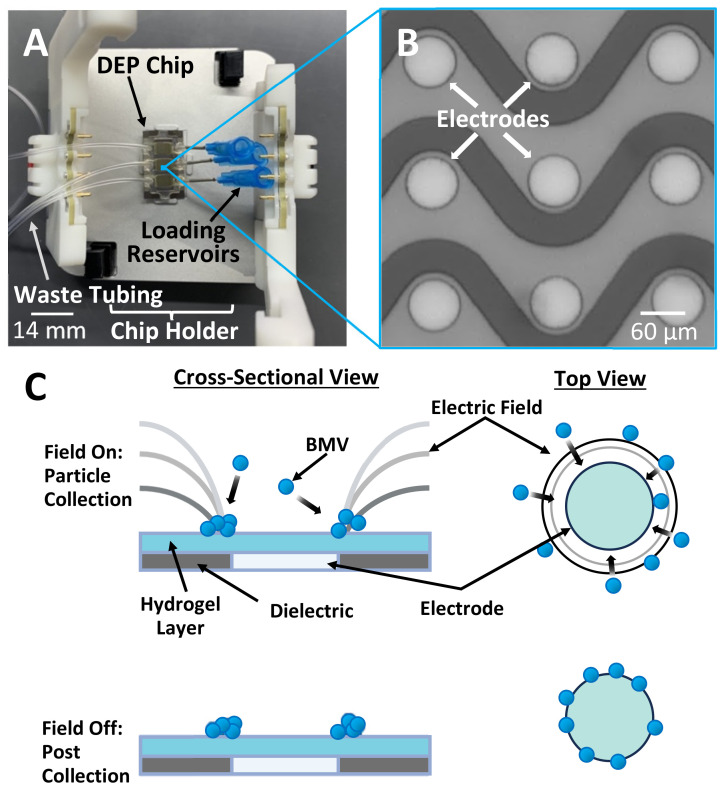
Overview of dielectrophoresis (DEP)-based bacterial membrane vesicle (BMV) collection on an electrokinetic chip. (**A**) A photo of the electrokinetic chip setup used for particle collection inside a specially designed holder that stabilizes the chip and makes electrical connections to the microelectrode array. The chip contains fluidic channels each with an array of microelectrodes. (**B**) A small subsection of the electrode array within the electrokinetic chip showing the platinum circular electrodes. (**C**) Schematic diagram showing both cross-sectional and top views of an electrode from the array during DEP collection and after collection.

To address the challenges of recovering BMVs from plasma, we have developed a new method that utilizes dielectrophoresis (DEP)-based forces which take advantage of differences in the dielectric properties between the particles and the surrounding fluid and have been shown to rapidly recover particles similar to BMVs from complex mixtures [16,17,18]. DEP force generation can be performed using microelectrode arrays integrated into microfluidic devices, which require as little as 20 µL of sample volume (Figure 1A,B). The devices used in this experiment have an array of microelectrodes which are designed to generate electric field gradients that result in electric field factors strong enough to capture sub-micron particles from high-conductance samples by applying an alternating electric field (Figure 1C). The electric field generated on these devices has been extensively modeled and proven capable of generating electric field gradients strong enough to isolate sub-micron particles [19,20]. The full experimental setup for applying the electric field to the devices and subsequent imaging can be seen in the Supplementary Materials (Supplementary Figure S1).

By adjusting the frequency of the applied alternating electric field, a subset of nanoparticles can be selectively isolated from the fluid by drawing them to the region around the electrode edge where the electric field factor is the highest (Figure 1C). Previous experiments have demonstrated that a frequency of 14 kHz is optimal for isolating nanoparticles, including extracellular vesicles and liposomes [21,22,23,24]. Simultaneously, larger particles and fragments of cellular debris are pushed away from regions with a high electric field factor. The DEP force ($F_{DEP})$ on a given particle can be predicted using the classical DEP equation:

(1) $$F_{DEP}=2\pi \epsilon_{m}R^{3}R\left[CM^{*}\right]\nabla {\left|E\right|}^{2}$$

where $\epsilon_{m}$ is the electric permittivity of the media, *R* is the radius of the particle, $R\left[CM^{*}\right]$ is the real part of the frequency dependent Clausius–Mossotti factor, and $\nabla {\left|E\right|}^{2}$ is the electric field factor [25]. DEP has successfully been used to collect and analyze many different types of biological particles, including exosomes, live bacteria, cell-free DNA, viruses, and circulating tumor cells [22,26,27,28]. DEP can recover particles from high-conducting buffered solutions and from complex high-conducting fluids such as whole blood, plasma, and undiluted model saliva [22,26,27]. DEP is advantageous because it does not require labeling of the desired particles prior to isolation, which streamlines the isolation protocol and allows for observations of particle properties in their native form.

Another benefit of DEP recovery is that it concentrates the collected particles around the electrode edge. This increases the signal-to-noise ratio, resulting in a greater level of sensitivity compared to UC where the recovered particles need to be resuspended in a wash buffer for recovery and analysis, resulting in particle dilution. Another advantage of DEP is that the particle collection zone is spatially well defined, which is critical for on-chip biomarker quantification through fluorescence staining, something that is not possible with UC. Finally, the DEP method is highly parallelizable and could result in higher levels of throughput than UC.

The BMVs recovered by DEP are co-collected with other nanoparticles of a similar size and material composition, such as eukaryotic extracellular vesicles. This is also the case with UC, where particles with a similar size and buoyant density are co-collected. We demonstrate here that the DEP-recovered BMVs are of sufficient purity to allow for successful immunostaining and detection of bacteria-specific biomarkers. Despite rapidly rising interest in using BMVs for diagnostic applications, the isolation and analysis of BMVs via DEP has not been previously demonstrated.

Herein, we demonstrate the isolation of BMVs from human plasma via DEP performed on an electrokinetic chip. We show that collected BMVs can be analyzed further for bacterial classification via immunostaining and quantified on-chip, which streamlines the analysis process and eliminates any biomarker loss that could occur through fluid transfers. The information provided by recovering BMVs via DEP and analyzing them on-chip demonstrates the potential for using DEP in point-of-care and in-the-field applications by circumventing the large equipment and time-consuming and labor-intensive methods of BMV recovery currently available.

## 2. Methods

### 2.1. Materials

BL21 *Escherichia coli* were purchased from New England Biolabs (Ipswich, MA, USA C2527H). Difco LB Broth, Miller (Luria–Bertani) (Fisher Scientific, Hampton, NH, USA) (244610, lot #1285538), Gibco Dulbecco’s phosphate-buffered saline (14190-144, lot #2717594), glutaraldehyde 50% aqueous solution (A10500-0E, lot #10242942), bovine serum albumin (B14, 2975507), Vybrant DiO cell-labeling solution (V22886, lot #2491432), and Invitrogen donkey anti-goat IgG (H + L) cross-absorbed secondary antibody, AlexaFluor647 (A21235, lot #2482945), were all purchased from Thermo Fisher Scientific (Waltham, MA, USA). The Millipore Stericup quick-release vacuum filtration systems of 0.45 µm pore size and 500 mL capacity units were purchased from Millipore Sigma (Dramstadt, Germany; S2HVU05RE, lot #MP224708G2). Amicon stirred cells, 200 mL, were purchased from Millipore Sigma (Darmstadt, Germany; UFSC20001). Ultracel 30 kDa ultracentrifugation disks were purchased from Millipore Sigma (Darmstadt, Germany; PLTK07610, lot #C3BB90755). Goat anti-lipid A LPS antibody was purchased from Bio Rad (Hercules, CA, USA, OBT1844, lot #163258). Polyclonal rabbit anti-CD9 antibodies and goat anti-rabbit HRP secondary antibodies for Western blot analysis were purchased from System Biosciences Innovation (Palo Alto, CA, USA; EXOS-CD9A-1, lot#190911-002, dilution 1:1000; EXOSAB-HRP, lot#190408-015, dilution 1:20,000). Donkey anti-goat HRP antibodies were purchased from Promega Corporation (Madison, WI, USA; V805A, lot#547026, dilution 1:5000). SuperSignal Pico PLUS Chemiluminescent Substrate and Pierce BCA Protein Assay Kits were purchased from Thermo Scientific (Waltham, MA, USA; 34580, 23225). BD Difco skim milk was purchased from Midland Scientific (Omaha, NE, USA; 232100). Bolt Sample Reducing Agent was purchased from Invitrogen (Waltham, WA, USA; B0009). Formvar/carbon 300 mesh support grids were purchased from Ted Pella Inc. (Redding, CA, USA; 01753). Uranyl acetate was purchased from Electron Microscopy Sciences (Hatfield, PA, USA; 22400, lot #170809-03).

De-identified pooled human plasma (blood derived) was purchased from Innovative Research (Novi, MI, USA; lot #HMN923275) which obtained the plasma from consented donors as part of an IRB. All experimental protocols were carried out in accordance with relevant guidelines and regulations outlined by the OHSU Institutional Review Board.

### 2.2. Preparation and Characterization of Bacterial Membrane Vesicles

#### 2.2.1. Isolation of Bacterial Membrane Vesicles from Culture

**Figure 2:**
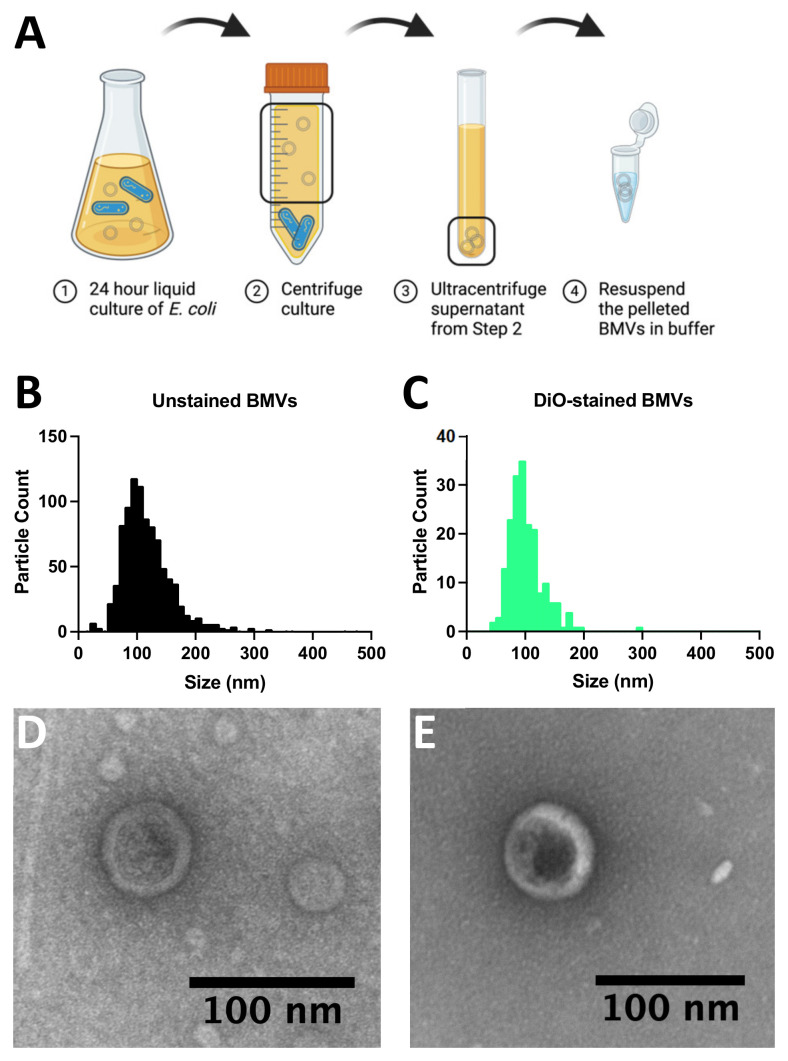
Characterization of bacterial membrane vesicles (BMVs). (**A**) Schematic diagram showing the collection of BMVs from BL21 *Escherichia coli* cultures. (**B**) Size distribution of *E. coli* BMV diameters determined using nanoparticle tracking analysis. (**C**) Nanoparticle tracking analysis of the diameter distribution of DiO-stained *E. coli* BMVs. (**D**) Transmission electron microscope image of negative-stained *E. coli* BMVs. (**E**) Transmission electron microscope image of negative-stained *E. coli* BMVs also stained with DiO.

An overnight culture of BL21(DE3) *Escherichia coli* grown in Luria–Bertani broth (LB) was diluted 1:1000 into 1 L of fresh LB and grown for 24 h at 37 °C, 250 RPM (Figure 2A). The culture was then centrifuged for 45 min at 3000× *g* at 4 °C, and the supernatant was poured off and filtered through a 0.45 µm filter (Figure 2A). The filtered supernatant was then condensed in an Amicon filtration unit with a 10kDa filter. The condensed supernatant was ultracentrifuged at 150,000× *g* for 3 h at 4 °C. The supernatant was poured off, and the BMV pellet was resuspended in sterile 1x PBS and then filtered through a 0.45 µm filter. The purified BMVs were stored at 4 °C for a maximum of one week, or frozen at −80 °C if they were to be used more than a week from collection.

#### 2.2.2. Nanoparticle Tracking Analysis of Bacterial Membrane Vesicles

Size distribution and concentration measurements of the BMVs were obtained using a ZetaView PMX-420 nanoparticle tracking analysis system from Particle Metrix (Inning am Ammersee) (Starnberg, Germany). For each measurement, 11 fluid cell positions were scanned at 30 frames/s with a shutter setting of 100 using a 488 nm laser at 40 mW. The cell temperature was regulated at 25 °C. Analysis was performed using the ZetaView software version 8.05.12 SP2. Samples were diluted 1:1000 in 0.5X PBS before each measurement.

#### 2.2.3. Transmission Electron Microscopy

Purified BMVs were blotted onto glow-discharged 300-mesh formvar/carbon-coated copper grids. The grid-adhered BMVs were then incubated with goat anti-lipopolysaccharide (LPS) antibody (Bio Rad, lot #163258) (Hercules, CA, USA) diluted 1:10 in 3% BSA in PBS and subsequently incubated with anti-goat antibody conjugated to 10 nm gold beads diluted 1:8 in 3% BSA in PBS. Samples were then fixed with 2% glutaraldehyde and negative-stained with 0.4% uranyl acetate. Imaging of the grids was performed at 120 keV on a Tecnai Spirit TEM system (FEI Company, Hillsboro, OR, USA) using the AMT software interface, AMT Capture Engine v7.00, on a NanoSprint12S-B CMOS camera system (AMT).

#### 2.2.4. Western Blot Analysis

The protein concentration of samples containing isolated Henrietta Lacks (HeLa) cell line-derived EVs and BL21 *Escherichia coli* BMVs were determined using the BCA assay and then normalized to equal concentrations of 4.33 μg/mL. Normalized samples were lysed by the addition of 30% NP-40 with protease inhibitors, set on ice for 30 min, and then passed through a 30G needle up to 6 times. Samples were then reduced by DTT and then incubated in 95 °C for 5 min. Samples were then loaded into a 4–12% Tris-Bis gel and run for 22 min at 200V in 1xMEPS buffer. Mini Blot Module was then used to transfer protein to a 0.45 μm PVDF membrane by applying 20 V for 1 h. Protein-transferred membranes were blocked in 5% fat-free milk in 1xTBST for 1 h and then incubated with primary anti-lipid A LPS and anti-CD9 antibodies in 5% fat-free milk in 1xTBST with agitation in 4 °C overnight. The membranes were washed three times with 1xTBST, incubated with secondary HRP-conjugated antibodies in 5% fat-free milk in 1xTBST for 1 h at room temperature, and then washed three times with 1xTBST. Membranes were developed using chemiluminescent substrate and imaged using the iBright imaging system.

### 2.3. Dielectrophoresis Isolation and Analysis of Bacterial Membrane Vesicles

#### 2.3.1. Dielectrophoresis Isolation of Bacterial Membrane Vesicles

An aliquot of BMVs prepared in Section 2.2.1 was thawed and diluted 1:10 in pooled healthy human plasma. The BMV-spiked plasma solution was introduced to three- or eight-channel ExoVerita microfluidic chips purchased from Biological Dynamics (San Diego, CA, USA). Each channel of the microfluidic chips holds approximately 20 µL of sample and has 780 electrodes each 60 µm in diameter. A 25 µL sample of plasma was loaded into the inlet ports of the chip and a KD Scientific syringe pump was used to flow in the sample at a rate of 7 µL/min to a total volume of 20 µL. Once all channels were filled with the sample, the flow was stopped, and an alternating electric signal was applied. The electric signal was generated using an Agilent waveform generator with impedance set to High Z and a Newtons4th Ltd., Leicester, UK amplifier set to AC coupling, low bandwidth, and 4X amplification to apply a signal of 10 V_pp_ at 14 kHz. This signal was applied to the chip for a duration of 10 min. After collection, the applied alternating current was turned off and the microfluidic channel was rinsed with 50 µL of 0.5X PBS at a flow rate of 7 µL/min to remove uncollected particles.

#### 2.3.2. Immunofluorescence Staining of Dielectrophoresis-Isolated Bacterial Membrane Vesicles

After DEP recovery, the applied voltage signal was turned off for the remainder of the experiment and a 100 µL blocking solution of 2% milk (in 0.5X PBS) was loaded into the inlet ports and a syringe pump was used to flow in the solution at a rate of 7 µL/min to a total volume of 50 µL. The solution was incubated on-chip for 20 min and then a wash step of 50 µL of 0.5X PBS buffer at a flow rate of 7 µL/min rinsed away the remaining milk. The collected BMVs on-chip were stained for 1 h with goat anti-lipid A LPS antibody suspended in 2% milk (1:200). After incubation, a wash step of 50 µL of 2% milk at a flow rate of 7 µL/min rinsed away all the unbound antibodies. The chip was then incubated for 1 h with Alexa Fluor 647 donkey anti-goat antibody suspended in 2% milk (1:500). After incubation, a wash step of 50 µL 0.5X PBS at a flow rate of 7 µL/min rinsed away the unbound antibody and 2% milk. After rinsing, fluorescence imaging was performed using a Zeiss Axio Imager Vario A2 microscope (Zeiss, Oberkochen, Germany).

#### 2.3.3. Scanning Electron Microscopy

A microfluidic chip was used to collect BMVs, as described in Section 2.3.1. The chip was then washed with DI water and stored in a −80 °C freezer overnight to freeze the fluid. The cold temperature also allowed for the removal of the glass covering and adhesive tape, leaving the frozen water over the electrode array exposed. The chip was then lyophilized to remove the frozen water to expose the collected BMVs. The array was then adhered to an 18 mm aluminum pin-style SEM stub with carbon tape. A Leica EM ACE600 High Vacuum Sputter Coater (Leica Microsystems, Wetzlar, Germany) equipped with a planetary stage was used to conductively coat the surface with 8 nm carbon. SEM imaging was performed on a Thermo Scientific Helios UC5 NanoLab DualBeam SEM (Thermo Fisher Scientific, Waltham, MA, USA) using a 1 keV and 13 pA beam, 4 mm working distance, and the TLD detector (TLD, New York, NY, USA) in secondary electron mode. Images were collected at both 0° and 45° stage tilt.

#### 2.3.4. Statistical Analysis

To assess system variability, three replicates were performed using the protocol described in Section 2.3.1 and Section 2.3.2 staining with either an anti-LPS antibody for BMVs isolated from plasma or a lipid intercalating dye, DiO for BMVs, isolated from 0.5X PBS. The stained collected material was quantified using a custom MATLAB program [21]. The coefficient of variation was calculated by dividing the standard deviation by the mean for each staining method. A negative control was also performed by applying the DEP parameters to a plasma sample without added BMVs. GraphPad Prism v9.4.0 was used to generate the bar graphs and conduct a Student’s two-tailed unpaired *t*-test where **** represents a *p* value less than 0.0001. Lower limit of detection (LLD) analysis was performed using GraphPad Prism v9.4.0. The LLD value was determined by measuring the mean background fluorescence signal and adding 3 times the standard deviation ($\bar{x}$ ± 3 × SD). Then, the line of best fit for the BMV fluorescence dilution curve was calculated and the intersection with the LLD was determined.

## 3. Results and Discussion

To demonstrate the recovery of bacterial membrane vesicles (BMVs) using dielectrophoresis (DEP), BMVs were first isolated from BL21 *Escherichia coli* cultures using standard procedures (Figure 2A) and characterized via nanoparticle tracking analysis and transmission electron microscopy (Figure 2B–E). BMVs were isolated from a liquid bacterial culture via ultracentrifugation and stored at 4 °C if used within one week, or frozen at −80 °C if they were to be used more than a week from collection (Figure 2A). The size and concentration of purified BMVs were assessed using nanoparticle tracking analysis by observing both unlabeled particles (Figure 2B) and BMVs labeled with a lipid intercalating dye (DiO) (Figure 2C). The mean particle diameter for unlabeled particles was 127 ± 54 nm with a concentration of 4.7 × 10^10^ particles/mL. The stained BMVs showed a mean particle size of 102 ± 31 nm with a concentration of 1.4 × 10^10^ particles/mL. The presence of purified BMVs was further confirmed via transmission electron microscopy, which shows circular particles around 100 nm in diameter in samples of BMVs unlabeled (Figure 2D) and labeled (Figure 2E) with DiO.

BMVs purified from *E. coli* culture and labeled with DiO were then isolated using DEP. An alternating electric current frequency of 14 kHz was applied to the electrode array to successfully collect BMVs. This frequency was selected due to previous research showing its use to collect extracellular vesicles, which are eukaryotic analogs of BMVs with similar sizes and dielectric properties [21,22,23,24].

**Figure 3:**
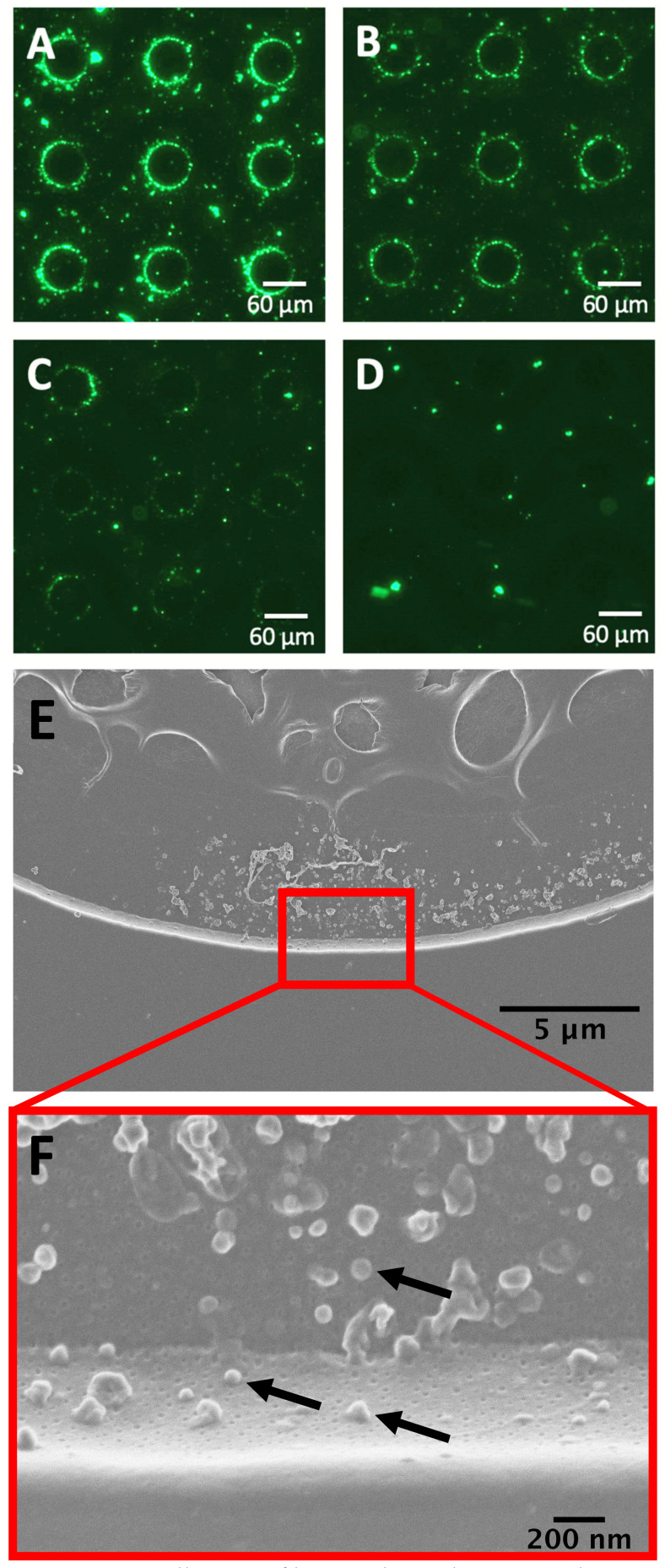
Dielectrophoresis (DEP) collection of bacterial membrane vesicles (BMVs). DEP collection of DiO-stained BMVs from 0.5x PBS with accumulation around the electrode edges at three different concentrations (**A**) 7 × 10^9^ BMVs/mL, (**B**) 4.7 × 10^9^ BMVs/mL, and (**C**) 2.8 × 10^9^ BMVs/mL. (**D**) The 500 nM solution of DiO in 0.5x PBS after DEP collection with no BMVs in solution as a control condition. (**E**) Scanning electron micrographs of a single electrode upon which unlabeled BMVs have been collected from 0.5x PBS. (**F**) Higher magnification image of panel E wherein individual BMVs can be visualized. Each particle is an isolated BMV with a few example BMVs indicated by arrows. The lower BMVs in the image are located on the vertical edge of the electrode, showing their vertical profile. The BMVs above them are on the flat electrode surface and are viewed from the top down. Control SEM images in Supplementary Figure S2 show that no background particles were collected from the purified buffer.

BMVs stained with DiO were diluted 1:1, 1:2, and 1:4 in 0.5X PBS to concentrations of 7 × 10^9^ BMVs/mL, 4.7 × 10^9^ BMVs/mL, and 2.8 × 10^9^ BMVs/mL, respectively, and isolated using DEP on the electrokinetic microfluidic device (Figure 3A–C). The collection of fluorescently labeled particles was observed around the edge of each electrode. Representative images of a nine-electrode subsection from each array are shown. The fluorescence intensity around the electrode edges decreased with the decreasing concentration of BMVs. To determine the background level of aggregated DiO collection, a control solution of 500 nM DiO in 0.5X PBS was exposed to the same alternating electric field as the labeled BMVs, but no collection around the electrode edges was observed (Figure 3D). To further validate the isolation of BMVs using DEP, scanning electron microscopy of an electrode edge was performed to visualize the particles (Figure 3E,F). BMVs are visible in profile collected along the vertical edge around the electrode and from top down on the flat electrode surface. A few of the BMVs are denoted by black arrows. DEP was also applied to a control chip containing only 0.5x PBS, and the chip was then imaged using scanning electron microscopy to confirm that no nanoparticles were collected from the buffer solution (Supplementary Figure S2).

**Figure 4:**
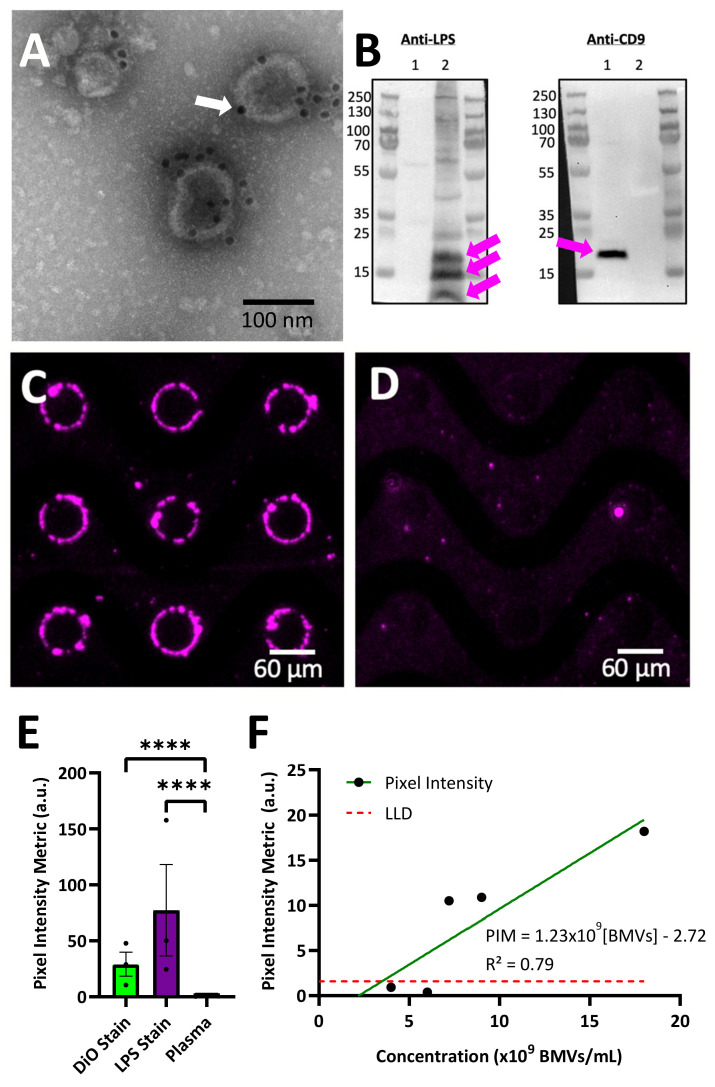
On-chip collection and immunostaining of bacterial membrane vesicles (BMVs) from human plasma. (**A**) Transmission electron microscope image of immunogold-labeled *Escherichia coli* BMVs. BMVs are labeled with a goat anti-lipid A lipopolysaccharide (LPS) primary antibody and an anti-goat 10 nm gold bead-conjugated secondary antibody. Lipid A is a component of LPS, a Gram-negative bacterial marker. An example of an individual gold bead is indicated by the white arrow. (**B**) Western blots showing HeLa EVs (Lane 1) and *E. coli* BMVs (Lane 2) stained for LPS, and the EV marker CD9. The EVs were positive for CD9 but not LPS, and the BMVs were positive for LPS but not CD9. Positive staining bands are indicated with magenta arrows. The BMVs have 3 main positive staining bands for LPS which could be due to the lipid A component of LPS being attached to varying lengths of the larger LPS molecule. (**C**,**D**) dielectrohporesis (DEP) collection of nanoparticles and immunofluorescent staining for LPS, a bacteria-specific marker, from (**C**) human plasma with a 1:4 dilution of *E. coli* BMVs and (**D**) an unaltered human plasma sample control. (**E**) A bar graph showing three replicates of DEP isolation of BMVs isolated from buffer stained with a lipid intercalating dye (DiO), spiked BMVs isolated from plasma stained with a fluorescently labeled antibody against LPS, and plasma without BMVs collected under the same conditions as a negative control (n = 3, mean ± standard error of the mean, Student’s *t*-test, **** *p* < 0.0001, α = 0.05). (**F**) A dilution curve of BMVs diluted in human plasma, recovered using DEP, and quantified on-chip. Recovered BMVs were stained with goat anti-LPS primary antibodies followed by anti-goat AlexaFluor 647 secondary antibodies. The lower limit of detection (LLD) is shown on-graph as 4.31 × 10^9^ BMVs/mL. The R-squared value of the linear fit of the dilution curve is 0.79, which suggests a linear correlation.

To our knowledge, this is the first demonstration of BMV isolation using DEP. To demonstrate the versatility of this technique to collect BMVs from high-conducting complex body fluids, BMVs were isolated from human plasma (Figure 4). A stock of purified unlabeled BMVs were spiked into pooled healthy human plasma samples. To specifically label BMVs after DEP collection from plasma, an antibody against lipid A lipopolysaccharide (LPS), which is a Gram-negative bacteria-specific biomarker found in BMVs and not expected to be found in healthy human plasma, was used. Validation of the antibody used for immunolabeling was performed using transmission electron microscopy and Western blot analysis. For transmission electron microscopy (Figure 4A), purified BMVs were stained with the primary antibody against LPS and then stained with a secondary antibody labeled with 10 nm gold particles (dark circles), confirming that LPS was present on the surface of the BMVs as expected. A control sample of BMVs stained with just the gold-conjugated secondary antibody confirmed an absence of non-specific staining (Supplementary Figure S3). A Western blot analysis was used to further confirm the presence of LPS on the BMVs and absence of LPS on human-derived nanoparticles (Figure 4B). The Western blot revealed that BMVs stained positive for LPS, as expected when derived from Gram-negative parent bacteria, while human-derived extracellular vesicles (EVs) isolated from the human HeLa cell line did not stain positive. Multiple bands staining positive for LPS can be seen in the BMV sample, likely corresponding to the naturally varying lengths of the polysaccharide chain portion of LPS. Both EVs and BMVs were also stained with an antibody against CD9, an EV surface marker that is not present on BMVs. The isolated BMVs did not stain positive for CD9, while the EVs did stain positive. Uncropped Western blot images can be seen in Supplementary Figure S4. This analysis reveals that the LPS antibody stains LPS-containing BMVs and does not stain human EVs.

Unstained BMVs were spiked into a human plasma sample to a final concentration of 1.8 × 10^10^ BMVs/mL and were successfully recovered using DEP. The collected material around the electrode edge, which was expected to contain a mixture of BMVs along with endogenous EV particles, was stained with the anti-LPS antibody, and BMVs were observed around the electrode edge via fluorescence imaging (Figure 4C). To further validate the DEP collection of BMVs, a plasma sample without spiked BMVs was run on the DEP chip under the same conditions and stained with the anti-LPS antibody (Figure 4D). No fluorescence signal was observed around the electrode edge, showing that the signal in Figure 4C was from collected BMVs.

To understand the reproducibility of DEP-based BMV collection, multiple replicates of collection and staining were performed with two different staining methods. The first was three replicates of BMV isolation from 0.5X PBS at a concentration of 4.5 × 10^9^ BMVs/mL stained with the lipid intercalating dye DiO. The second was three replicates of spiked BMV isolation from human plasma at a concentration of 4.5 × 10^9^ BMVs/mL stained with an antibody against LPS (Figure 4E). The coefficient of variation for the three replicates was found to be 0.52 for staining BMVs isolated from buffer using DiO and 0.74 for staining BMVs isolated from undiluted plasma using an anti-LPS antibody. This shows reproducible BMV collection by DEP from both buffer and plasma. Variability can be reduced through adjustments to experimental parameters and the use of internal standards to adjust for chip-to-chip variability.

The lower limit of detection of BMVs from a human plasma sample was determined by a serial dilution of BMVs into plasma samples followed by DEP isolation and on-chip LPS staining. The lower limit of detection was determined to be 4.31 × 10^9^ BMVs/mL (Figure 4F).

DEP collection of BMVs from plasma took 10 min followed by a 10 min wash for purification. The on-chip immunostaining process took an additional 3 h, 2 of which consisted of incubation with the staining antibodies. This long staining time was chosen to ensure full staining of the collected BMVs but could be optimized to a shorter time period. Overall, the DEP technique required considerably less time and labor compared to the traditional ultracentrifugation method where just BMV recovery from a buffer can take over 3 h [15,30,31] and even longer when recovering from higher-viscosity fluids like plasma [32]. To immunostain the ultracentrifuged BMVs, the stain needs to be introduced and the particles washed, requiring additional 3 h ultracentrifugation sessions, resulting in a total time of 12 h to produce purified and stained BMVs. Sample loss is also high during these ultracentrifugation washes, a problem not encountered by the DEP technique since the BMVs are held at the electrode edge, resulting in low levels of loss throughout the DEP washing steps.

## 4. Conclusions

BMV nanoparticles were successfully recovered from buffer and from high-conducting human plasma using the DEP microfluidic chip. These collected particles were then successfully quantified and classified as being derived from Gram-negative bacteria on the chip using immunostaining techniques for the Gram-negative bacteria-specific biomarker lipid A LPS. The lower limit of detection of these BMVs in plasma was found to be 4.31 x 10^9^ BMVs/mL, which is a useful range for sepsis diagnostics. To our knowledge, these experiments demonstrate the first use of DEP to recover BMVs. The isolation and purification of BMVs from a complex plasma sample using DEP can be performed in 20 min, and immunostaining can be performed in 3 h directly on the chip, which is much shorter than the 12 h required to isolate and stain BMVs from a plasma sample using the gold-standard method of ultracentrifugation followed by additional time for biomarker quantification analysis. Furthermore, the dielectrophoretic isolation of BMVs is easier to scale and automate compared to ultracentrifugation, which can enable high-throughput BMV analysis. These exciting results show that DEP technology is a valuable tool to enable the efficient collection and quantification of BMVs with the speed, ease of use, and high automation potential needed for the future clinical translation of BMV-based diagnostics.

## Data Availability

The data that support the findings of this study are available from the corresponding author upon reasonable request.

## Supplementary Materials

The following supporting information can be downloaded at: https://www.mdpi.com/article/10.3390/bios14100456/s1, Figure S1: Experimental Setup. Labeled photo showing the equipment used for all experimental procedures involving dielectrophoresis. Figure S2: Scanning electron microscopy control. [A] Scanning electron micrograph of a singular electrode upon which DEP has been performed on 0.5x PBS, showing an absence of collected material. DEP was performed on 0.5x PBS to confirm absence of collection around electrode edges. [B] Scanning electron micrograph of the edge of an electrode upon which DEP has been performed on 0.5x PBS. Figure S3: Transmission electron microscopy control. Transmission electron micrograph of immunogold stained *Escherichia coli* BMVs. BMVs were stained with an anti-goat 10 nm gold bead-conjugated secondary antibody only to confirm absence of non-specific binding. Figure S4: Full images of bacterial and mammalian marker analysis by western blot. HeLa EVs (lane 1) and BL21 *E. coli* BMVs were normalized for protein concentration and analyzed for [A] gram-negative bacteria marker lipid A LPS and [B] EV marker CD9.
