# Supplementary material for: Recovery and Analysis of Bacterial Membrane Vesicle Nanoparticles from Human Plasma Using Dielectrophoresis
Source: Biosensors (Basel). 2024 Sep 25;14(10):456. doi: 10.3390/bios14100456 (PMC11505931; doi:10.3390/bios14100456)
Supplement: Supplementary file 1 [file biosensors-14-00456-s001.zip › biosensors-3110696-supplementary.pdf]

Recovery and Analysis of Bacterial Membrane Vesicle Nanoparticles from Human Plasma using Dielectrophoresis

<u>Supplementary Information</u>

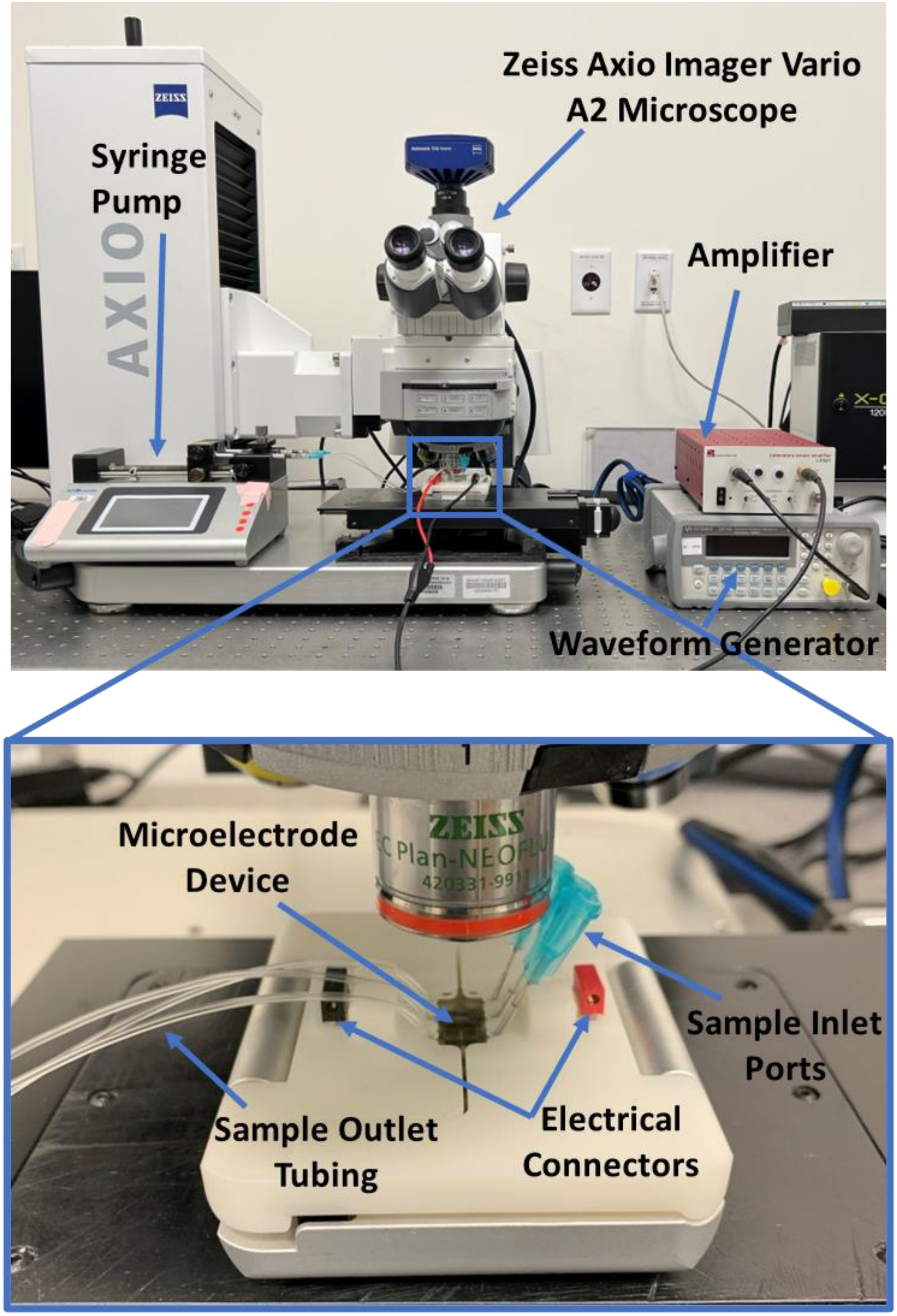

Supplementary Figure S1– Experimental Setup. Labeled photo showing the equipment used for all experimental procedures involving dielectrophoresis.

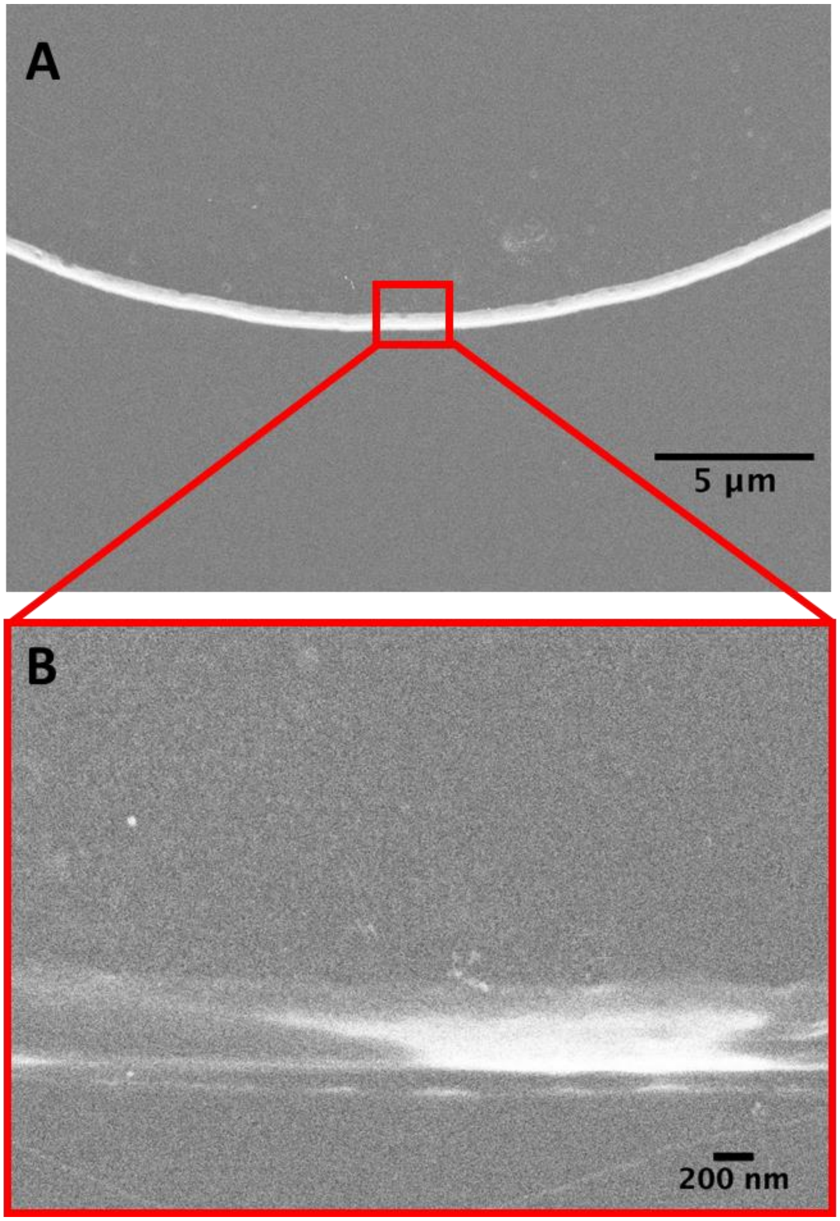

Supplementary Figure S2 – Scanning electron microscopy control. [A] Scanning electron micrograph of a singular electrode upon which DEP has been performed on 0.5x PBS, showing an absence of collected material. DEP was performed on 0.5x PBS to confirm absence of collection around electrode edges. **[B]** Scanning electron micrograph of the edge of an electrode upon which DEP has been performed on 0.5x PBS.

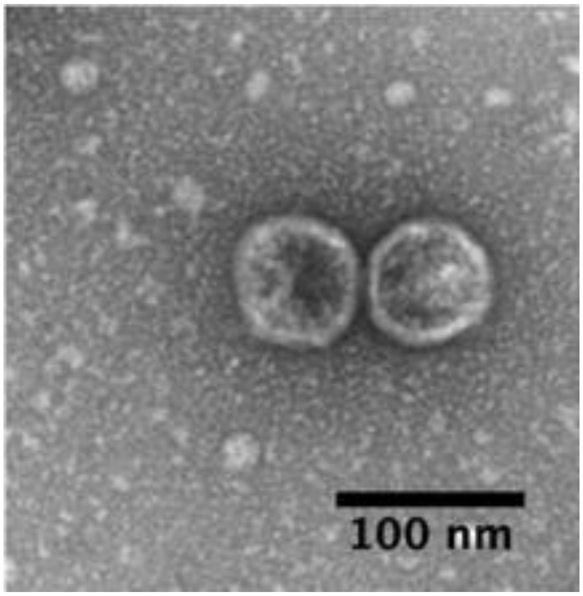

Supplementary Figure S3 – Transmission electron microscopy control. Transmission electron micrograph of immunogold stained *Escherichia coli* BMVs. BMVs were stained with an anti-goat 10 nm gold bead-conjugated secondary antibody only to confirm absence of non-specific binding.

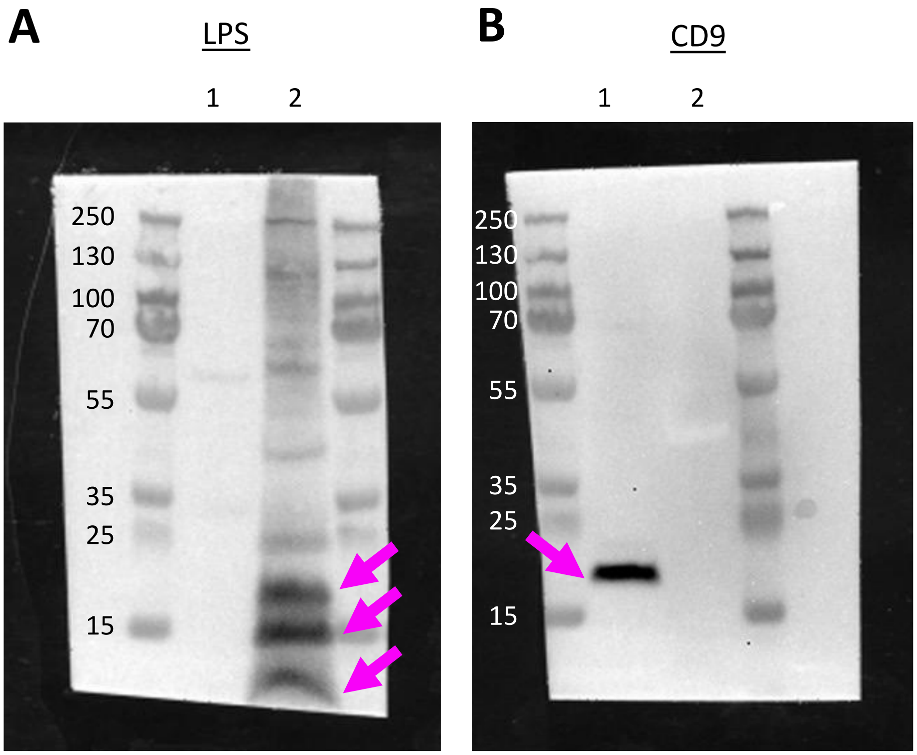

Supplementary Figure S4 – Full images of bacterial and mammalian marker analysis by western blot. HeLa EVs (lane 1) and BL21 *Escherichia coli* BMVs were normalized for protein concentration and analyzed for **[A]** gram-negative bacteria marker lipid A LPS and **[B]** EV marker CD9.
